# Infundibuloneurohypophysitis Associated With Sjögren Syndrome Successfully Treated With Mycophenolate Mofetil: A Case Report

**DOI:** 10.1097/MD.0000000000003132

**Published:** 2016-04-01

**Authors:** Camille Louvet, Salwan Maqdasy, Marielle Tekath, Vincent Grobost, Virginie Rieu, Marc Ruivard, Guillaume Le Guenno

**Affiliations:** From the Department of Internal Medicine (CL, VG, VR, MR, GLG) and Department of Radiology (MT), Centre Hospitalier Universitaire Estaing; and Department of Endocrinology and Diabetology (SM), Centre Hospitalier Universitaire Gabriel Montpied, Clermont-Ferrand, France.

## Abstract

Hypophysitis is an inflammatory disorder of the pituitary gland and corticosteroids are usually recommended as the first-line treatment. Hypophysitis related to primary Sjögren syndrome (pSS) is uncommon. We describe the unusual case of a patient with infundibuloneurohypophysitis associated with pSS successfully treated with mycophenolate mofetil (MMF).

We describe a case of a 60-year-old man with a medical history of pSS presented with central diabetes insipidus and panhypopituitarism. Magnetic resonance imaging (MRI) revealed a thickening of the pituitary stalk and intense enhancement of the posterior pituitary, pituitary stalk, and hypothalamus. We diagnosed infundibuloneurohypophysitis associated with pSS. Hormonal replacement was started immediately and MMF was introduced without corticosteroids.

After 9 months of treatment, MRI of the pituitary revealed a complete regression of the nodular thickening of the pituitary stalk, with normal enhancement and appearance of the pituitary. The pituitary axes had completely recovered, whereas the diabetes insipidus was partially restored.

Our findings suggest that MMF is an effective alternative to corticosteroids for the treatment of lymphocytic hypophysitis associated with an autoimmune disease. Furthermore, this report could contribute to extend the spectrum of the neurological and endocrinological manifestations of pSS.

## INTRODUCTION

Hypophysitis is an inflammatory disorder of the pituitary gland that is considered to be an autoimmune disease. Lymphocytic adenohypophysitis (LA) is the most common histopathological type, but lymphocytic infiltration can also affect the infundibulum; in this case, the condition is termed lymphocytic infundibuloneurohypophysitis. Most hypophysitis is idiopathic, but new variants have been reported. The management of LA is poorly established, but corticosteroids are usually recommended as the first-line therapy.^1^

Primary Sjögren syndrome (pSS) is an autoimmune disease in which the exocrine glands undergo progressive infiltration by lymphocytes and plasma cells.^2^ Approximately 5% to 25% of patients with pSS exhibit central nervous system manifestations.^3^ Hypophysitis related to pSS is uncommon, and only 2 cases of hypophysitis associated with pachymeningitis have been reported.^4,5^

Here, we describe the unusual case of a patient with infundibuloneurohypophysitis associated with pSS successfully treated with mycophenolate mofetil (MMF).

## CASE REPORT

A 60-year-old man with a medical history of diabetes, hypertension, and peptic ulcers was referred to the Department of Internal Medicine at our institute with salivary gland hypertrophy, dry eyes syndrome, hypergammaglobulinemia, and neutropenia in 2010.

The results of an enzyme-linked immunosorbent assay were positive for antinuclear antibodies at a titer of 1/640, anti-Sjögren-syndrome-related antigen A and B autoantibodies (SSA and SSB), and DNA antibody at a titer of 29 UI/mL (<10 UI/mL). His absolute neutrophil count was 0.45 × 10^9^/L, and the results of a bone marrow aspiration were normal.

Xerophthalmia, xerostomia, and salivary gland hypertrophy evoked Sjögren syndrome. Besides the presence of autoantibodies (SSA and SSB), the diagnosis was confirmed thanks to Schirmer test and salivary gland biopsy. The former revealed a severe xerophthalmia with moisture limited only to 2 mm at 5 min, while the later showed a focal lymphocytic sialoadenitis (grade 4 according to Chisholm classification) on the histopathological study of the biopsy.^6^

Dryness was successfully treated by oral pilocarpine.

**FIGURE 1:**
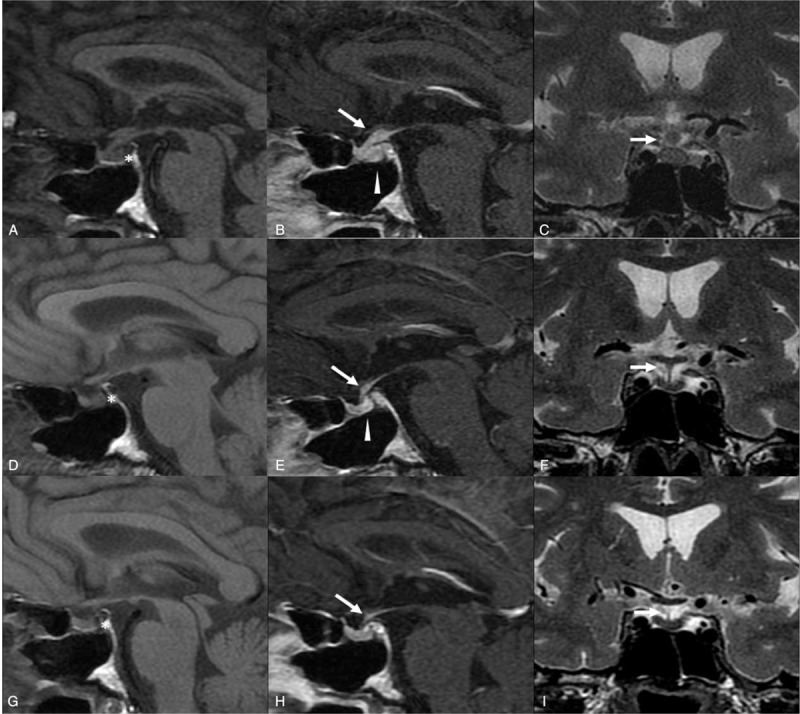
Magnetic resonance imaging (MRI) sagittal T1-weighted findings before (A, D, G) and after (B, E, H) gadolinium injection and coronal T2-weighted images (C, F, I). (A–C) Images captured in December 2013 before mycophenolate mofetil (MMF) therapy, showing loss of spontaneous T1 hyperintensity in the posterior pituitary (^∗^) associated with thickening of the pituitary stalk (white arrow). Intense enhancement of the posterior pituitary (arrow head) is evident in the pituitary stalk and hypothalamus. (D–F) MRI findings in June 2014 after 3 mo of MMF therapy, showing partial regression of the nodular thickening of the pituitary stalk (white arrow) and persistent enhancement of the posterior pituitary (arrow head). (G–I) MRI findings in January 2015 after 10 mo of MMF therapy, showing persistent loss of spontaneous T1 hyperintensity in the posterior pituitary (^∗^) and complete regression of the nodular thickening of the pituitary stalk (white arrow). The pituitary stalk exhibited normal enhancement and a normal appearance.

In December 2013, he complained of intense physical asthenia, anorexia, depression, and polydipsia and polyuria (5 L/d). A clinical examination revealed low blood pressure and a painless conjunctival infection. Laboratory hormonal findings confirmed panhypopituitarism with profound central hypogonadism, hypothyroidism, growth hormone deficiency, mild corticotropic insufficiency associated with hyperprolactinemia, and diabetes insipidus (Table 1). Other laboratory tests identified neutropenia (0.684 × 10^9^/L). Computed tomography of the thorax and abdomen revealed no abnormalities indicative of sarcoidosis or a tumoral process. The results of tests on the cerebrospinal fluid were normal. Hormonal replacement with hydrocortisone 15 mg/d, l-thyroxine 25 μg/d, and desmopressin 180 μg/d was started immediately, which had a dramatic effect on his asthenia and diabetes insipidus. Pituitary magnetic resonance imaging (MRI) revealed a loss of spontaneous T1 hyperintensity of the posterior pituitary, associated with thickening of the pituitary stalk and intense enhancement of the posterior pituitary, pituitary stalk, and hypothalamus (Figure 1A–C). We diagnosed infundibuloneurohypophysitis associated with pSS. MMF 2 g/d was introduced in March 2014.

**TABLE 1:**
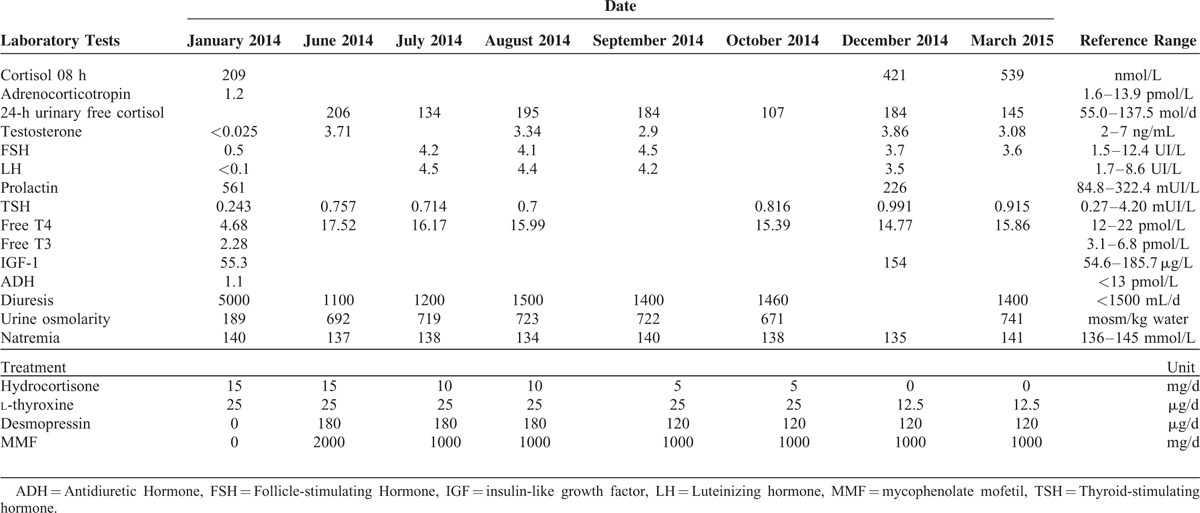
Hormonal Laboratory Test Results and Follow-Up Treatment

In June 2014, a clinical examination identified a slight decrease in salivary hypertrophy. Gonadotropic function had completely recovered, whereas pituitary corticotropic function was partially restored. Autoimmune neutropenia had improved (1.58 × 10^9^/L). MRI of the pituitary showed regression of the nodular thickening of the pituitary stalk, with persistent enhancement of the posterior pituitary (Figure 1D–F). These radiological and biological improvements necessitated a reduction in the dose of hydrocortisone to 10 mg/d and that of MMF to 1 g/d. In September 2014, the dose of hydrocortisone was further reduced to 5 mg/d because of high free urinary cortisol (195 nmol/d), and that of desmopressin was reduced to 120 μg/d because of hyponatremia and normal diuresis.

In December 2014, hydrocortisone was stopped, as early-morning cortisol level was normalized (421 nmol/L) and the free urinary cortisol was 184 nmol/d (normal levels: 27–325 nmol/d). Insulin-like growth factor-1 and prolactin levels normalized, and thyroid-stimulating hormone levels increased (Table 1).

In January 2015, MRI of the pituitary revealed a complete regression of the nodular thickening of the pituitary stalk, with normal enhancement and appearance of the pituitary.

During all the treatment by MMF, patient had good tolerance with no adverse events such as opportunist infections, digestive disorders, or hematologic disorders.

## DISCUSSION

Hypophysitis is a rare disorder that is primarily idiopathic or secondary to the occurrence of adjacent para-sellar masses, systemic disease, or adverse reactions to medication. The main diagnostic challenge is differentiating rare cases of LA from more common pituitary tumors. A definitive distinction can be obtained via pathological examinations, such as surgical biopsy of the pituitary. However, a presumptive diagnosis can often be made on the basis of a combination of context, clinical features, endocrinological assessment, and imaging studies.^7^

In this case, we did not perform pituitary biopsy, because of many symptoms consistent with pSS-associated autoimmune LA: anterior pituitary deficiency associated with diabetes insipidus is uncommon in patients with pituitary adenoma, and the degree of adenopituitary impairment was disproportionate with the small extent of the pituitary mass evident on MRI. Thickening of the pituitary stalk, and enhancement of the posterior pituitary and hypothalamus were strongly suggestive of infundibuloneurohypophysitis. In addition, the efficacy of treatment with MMF confirmed a diagnosis of autoimmune LA.

To the best of our knowledge, isolated infundibuloneurohypophysitis in a patient with pSS has never been reported. Only 2 cases of pSS with hypophysitis have been reported associated with hypertrophic cranial pachymeningitis.^4,5^ The first case was that of a 73-year-old man with pSS associated with hypopituitarism and diabetes insipidus. MRI revealed extensive enhancement affecting the dura mater, hypothalamus, cavernous sinus, and pituitary gland and stalk. In this case, corticosteroid pulse therapy did not improve pituitary function. The second case was that of a 47-year-old woman with pSS, who developed right sensory neural hearing loss followed by right facial palsy. MRI revealed mild diffuse thickening and enhancement of the dura mater and enhancement of the pituitary gland. After corticosteroid therapy, follow-up MRI of the brain showed minimal regression of the pituitary gland enlargement and meningeal thickening.

The optimal treatment of LA is controversial. Some advocate reducing the inflammatory process, whereas others recommend replacement therapy alone. Mass reduction can be achieved by pituitary surgery, the administration of drugs (glucocorticoids, azathioprine, or methotrexate), or radiotherapy. The indications for surgery are restricted to the presence of deficits of the visual field, without any benefit for pituitary function. Glucocorticoids can be effective by reducing the size of the pituitary mass or thickened stalk. Kristof et al^1^ performed the first and only prospective trial of the administration of glucocorticoids in 9 patients with lymphocytic hypophysitis. They showed that methylprednisolone improved pituitary function in 4 patients and MRI findings in 8 patients after 1.5 to 6 months of treatment. Considering the number of patients and the absence of a control group, it is difficult to conclude that glucocorticoids are effective. Steroid therapy was not attempted in our case, because there were no signs of compression or visual disturbances, and steroids could have worsened the patient's diabetes mellitus. Other immunosuppressive drugs, such as azathioprine^8^ or methotrexate,^9^ have been used to treat LA. We chose MMF because it was safe and effective for the treatment of pSS, achieving a subjective improvement in ocular dryness and a significant reduction of hypergammaglobulinemia and rheumatoid factors.^10^ The efficacy of MMF in our case must be interpreted with caution because spontaneous partial or total recovery of pituitary function and mass resolution have been described in hypophysitis. Such cases represent only 3% of patients.^11^

## CONCLUSIONS

We report the case of a patient with isolated LA associated with pSS successfully treated with MMF. Our findings suggest that MMF is a useful alternative to corticosteroids in the management of patients with LA associated with autoimmune diseases. Furthermore, this report could contribute to extend the spectrum of the neurological and endocrinological manifestations of pSS.
